# Exploratory spatial analysis of autism rates in New York school districts: role of sociodemographic and language differences

**DOI:** 10.1186/s11689-020-09338-x

**Published:** 2020-12-16

**Authors:** Kathleen McGrath, Karen Bonuck, Mana Mann

**Affiliations:** 1 Albert Einstein College of Medicine, New York City, USA; 2 CUNY Graduate Center, New York City, USA

**Keywords:** Autism, English Language Learners, Geographic information systems, Sociodemographics, Disability

## Abstract

**Background:**

Literature on autism spectrum disorder (ASD) suggests *lower* ASD prevalence and *higher* age of diagnosis among children of color, from lower socioeconomic backgrounds, and from families with lower educational levels. These disparities have been attributed to factors such as limited access to diagnostic and treatment services, less opportunity for upward mobility to locales with ample resources, and linguistic barriers. However, few studies describe prevalence and geographic differences of ASD diagnoses by English Language Learner (ELL) status.

**Objectives:**

The primary objectives of this study are to (1) spatially explore the prevalence of ASD among New York State school districts and (2) examine differences of ASD prevalence rates between ELLs and native English-speaking peers.

**Methods:**

Using the 2016–2017 district-level data on public and non-public school age students (3–21 years old) receiving special education services in New York, we analyzed sociodemographic trends among school districts with varying percentages (low, medium, and high ranges) of students with ASD and ELLs. To do this, we conducted exploratory spatial analyses using GIS software, analysis of school district level demographic data, and multivariate linear regression.

**Results:**

In contrast to prior research on ASD prevalence among minority groups, we found disproportionately higher rates of ASD among school districts with higher proportions of Black and Hispanic students. Geographic analysis revealed statistically significant clustering of school districts with high ASD rates in New York City and Albany. Higher proportions of ELLs tended to be concentrated in densely populated, urban, and geographically smaller school districts and had higher proportions of Black, Hispanic, and Asian students.

**Conclusions:**

Schools with higher rates of ASD and ELL students tend to be concentrated in urban regions throughout New York and have higher representation of Black and Hispanic/Latino students, as well as higher rates of learning disabilities in general. Further research is warranted to explore possible reasons for this phenomenon.

## Background

Autism spectrum disorder (ASD) is a developmental disability present in early childhood and characterized by persistent impairments in social communication, restricted interests, and repetitive behaviors across multiple contexts [1]. Its prevalence in the United States has increased over the past few decades [2–5]. The Autism and Developmental Disabilities Monitoring (ADDM) Network, a nationwide surveillance system comprised of 14 clinical sites in the United States, reported an increase in the prevalence of ASD among 8-year-old children from 6.7 per 1000 in the year 2000 to 18.15 in 2016 [6–9]. Findings from the National Survey of Children’s Health (NSCH) and the National Survey of Children with Special Health Care Needs (NS-CSHCN), nationally representative parent-report surveys of U.S. children, also indicate a steady increase in ASD diagnoses over the past decade. Slightly higher than the ADDM Network findings, the prevalence rates reported from the NSCH and NS-CSHCN were 1–3% nationally [10–12].

The prevalence of ASD is reported to be *lower* and age of ASD diagnosis *higher* among children of color and families with lower educational levels and socioeconomic background. This is attributed to multiple factors, including limited access to diagnostic and treatment services and fewer opportunities for upward neighborhood mobility to locations with more resources [4, 10, 13–33]. Zuckerman and colleagues found that pediatric providers were less likely to screen Spanish (versus English) speakers for ASD and more uncomfortable with identifying ASD risk when families spoke Spanish [34]. In another report, Zuckerman and colleagues found that Latino parents with low English proficiency were more likely to experience barriers related to knowledge about ASD and trust in providers [35]. In addition, Latinos with Spanish as their primary language received less family-centered care for ASD than Caucasian families [27, 36].

Prior research has found an achievement gap between English Language Learner (ELL) and native English-speaking students, which may reflect a discrepancy between the student’s needs and services provided [37]. Schools play an important role in addressing disparities in ASD diagnosis by identifying and treating children who may otherwise be undiagnosed [30]. Blumberg et al. found that 20% of children with ASD were undiagnosed prior to starting primary school [10]. In addition, the Centers for Disease Control and Prevention surveillance data found that greater than 75% of ASD cases were identified through school systems, with school records being the sole record source for 40% of children [29].

School records may yield insights into potential disparities in diagnosis and services because they allow for geographic exploration of ASD prevalence among ELLs. Using data from New York State Department of Education, we aim to explore sociodemographic and geographic variations in ELL students and students with ASD in New York State. Our primary objectives are to (1) spatially explore ASD prevalence in New York State, utilizing data from school records and (2) identify differences in ASD prevalence between ELLs and native English-speaking peers, along with sociodemographic characteristics for each group. Through spatial and demographic analyses, we hope to provide an exploratory perspective of how language, race, and ethnicity may be associated with the geographic distribution of ASD in New York.

## Methods

In order to achieve our primary aims to (1) spatially explore ASD prevalence in New York State school districts and (2) identify differences in ASD prevalence among school districts with high versus low levels of ELL students, we first acquired school enrollment data on New York school districts for the 2016–2017 academic year. This dataset provided deidentified information on all New York public and non-public school students from pre-Kindergarten through Grade 12. This was the most recent data available that contained all variables needed for analysis. Because this data is geographically identified, but not spatially portrayed, we also needed a school district boundary line shapefile. This was acquired from the New York State GIS Program Office.

### Data sources

1. New York State Department of Education (NYS DOE): special education data collection and reporting unit, 2016-2017

As federally mandated by the Individuals with Disabilities Education Act (IDEA), all New York schools must provide annual reports on any students receiving special education services to the NYS DOE Special Education Data Collection and Reporting Unit. This includes all public, BOCES, Article 81, approved private, charter, Special Act, state supported, state acted, state agency, and out of state schools. This data is publicly available from the New York Department of Education’s online data portal.

- “Statewide totals as of October 4, 2016: by disability, county, and school district” [38]

This dataset provided the number of students (pre-K through Grade 12) with disabilities in every New York school district for the 2016–2017 academic year. This included the number of children who had been diagnosed with ASD, in addition to twelve additional IDEA categories (e.g., specific learning disability or speech or language impairment).

(b) “Statewide totals as of October 4, 2016: by enrollment, classification rate, and school district” [38]

This dataset provided the number of students (pre-K through Grade 12) enrolled in each school district for the 2016–2017 academic year. It was used to obtain an enrollment denominator to calculate ASD diagnosis per school district.

(c) “Enrollment database”, 2016 [39]

This dataset provided demographic information on students (pre-K through Grade 12) including gender, race/ethnicity, economic disadvantage, and English Language Learner status. Only data from the 2016–2017 academic year were used for this study.

(2) New York State GIS Program Office: NYS Schools and School District Boundaries, 2018 [40]

School district boundary shapefiles were obtained from the New York State GIS Program Office. These files provide school district boundaries for all K-12 schools (public, private, charter, and others) in New York State.

(3) United States Department of Education: Individuals with Disabilities Education Act data, 2016-2017 [41]

### Data conditioning and transformation

Data files were cleaned in Microsoft Excel to eliminate any excess variables or columns not needed for data analysis. Missing data was identified and eliminated from analysis. Because percentages of ASD and ELL status for small student populations could be potentially misleading, school districts with populations less than 150 students (*n* = 11) were removed before analysis. There were 31 New York City school districts in the dataset that were missing student population size. For this reason, student population data for these districts were individually obtained from the New York Department of Education data portal and manually entered into the data file. Because the original data did not include percentages (i.e., percentage of ELL, ASD, etc.), student population sizes were acquired from a separate data set. Percentage of learning disabilities, students with disabilities, ELLs, White, American Indian, Asian, Black, Hispanic, multiracial, and economically disadvantaged students were calculated in Microsoft Excel using student population as the denominator and multiplying by 100. This data was then imported into ArcMap 10.5.1 and merged via a spatial join to the New York school district shapefile.

### Data analysis

#### Table 1: sociodemographic characteristics of NY school districts stratified by ELL status

Descriptive summary statistics for all New York school districts (*n* = 895) included in analysis were calculated in ArcMap. Variables included percentages of ASD, ELL, racial/ethnic groups, economic disadvantage, learning disabilities, and overall disabilities (as identified by the Department of Education). School district data was then stratified into three tertiles (low, medium, and high) based on rates of English Language Learners. Descriptive summary statistics were then calculated in ArcMap for each group.

**Table 1:** Sociodemographic characteristics of NY school districts stratified by ELL status

	All school districts	Low ELLs (1st tertile)	Medium ELLs (2nd tertile)	High ELLs (3rd tertile)
*n* = 895	*M* (SD)			
Autism spectrum disorder^1^	1.14 (± 0.56)	1.08 (± 0.56)	1.08 (± 0.50)	1.26 (± 0.58)
English language learners^1^	2.59 (± 5.70)			
Male^1^	50.74 (± 5.15)	50.57 (± 4.97)	50.29 (± 6.86)	51.37 (± 2.67)
White^1^	78.27 (± 25.05)	91.82 (± 9.60)	87.88 (± 14.25)	55.11 (± 27.83)
Asian^1^	3.11 (± 5.97)	0.81 (± 1.09)	1.76 (± 2.35)	6.78 (± 8.94)
Hispanic^1^	9.84 (± 14.12)	2.81 (± 2.71)	4.23 (± 4.20)	22.49 (± 18.22)
Black^1^	5.39 (± 10.66)	1.46 (± 2.76)	2.16 (± 2.98)	12.59 (± 15.70)
American Indian^1^	0.40 (± 2.86)	0.55 (± 3.17)	0.45 (± 3.75)	0.21 (± 0.62)
Multiracial^1^	2.13 (± 1.96)	1.72 (± 1.59)	1.94 (± 1.78)	2.73 (± 2.29)
Economically disadvantaged^1^	44.50 (± 20.11)	47.72 (± 15.70)	42.15 (± 18.07)	43.63 (± 24.98)
Students with disabilities^1^	13.92 (± 4.93)	13.89 (± 4.21)	13.45 (± 4.67)	14.41 (± 5.75)
Learning disability^1^	5.21 (± 2.40)	5.60 (± 2.22)	5.12 (± 2.35)	4.91 (± 2.57)

^1^Percent of students per school district

#### Table 2: sociodemographic characteristics of NY school districts stratified by ASD rates

All sociodemographic and disability data (from Table 1) were then stratified into three new tertiles (low, medium, and high) based on rates of ASD per school district. Descriptive summary statistics were calculated in ArcMap for each group.

**Table 2:** Sociodemographic characteristics of NY school districts stratified by ASD rates

	All school districts	Low ASD rates (1st tertile)	Medium ASD rates (2nd tertile*)*	High ASD rates (3rd tertile)
*n* = 895	*M* (SD)			
Autism spectrum disorder^1^	1.14 (± 0.56)			
English language learner^1^	2.59 (± 5.70)	2.74 (± 7.61)	2.14 (± 3.76)	2.88 (± 5.00)
Male^1^	50.74 (± 5.15)	50.05 (± 8.47)	50.91 (± 1.89)	51.27 (± 1.86)
White^1^	78.27 (± 25.05)	80.93 (± 25.49)	79.55 (± 22.01)	74.33 (± 26.90)
Asian^1^	3.11 (± 5.97)	2.18 (± 4.85)	3.32 (± 6.07)	3.83 (± 6.72)
Hispanic^1^	9.84 (± 14.12)	8.59 (± 14.90)	9.45 (± 12.40)	11.49 (± 14.75)
Black^1^	5.39 (± 10.66)	3.50 (± 7.45)	5.07 (± 9.22)	7.60 (± 13.86)
American Indian^1^	0.40 (± 2.86)	0.64 (± 4.45)	0.30 (± 1.27)	0.27 (± 1.75)
Multiracial^1^	2.13 (± 1.96)	1.79 (± 1.96)	2.23 (± 1.83)	2.38 (± 2.04)
Economically disadvantaged^1^	44.50 (± 20.11)	44.06 (± 21.09)	42.50 (± 18.87)	46.94 (± 20.06)
Students with disabilities^1^	13.92 (± 4.93)	11.55 (± 5.36)	13.94 (± 3.03)	16.25 (± 4.91)
Learning disability^1^	5.21 (± 2.40)	4.6 (± 2.62)	5.11 (± 1.97)	5.85 (± 2.43)

^1^Percent of students per school district

#### Table 3: multiple linear regression of variables predicting ASD prevalence in New York school districts

A univariate ordinary least squares linear regression was conducted in R statistical software to evaluate the potential role of English Language Learner status as a predictor of ASD rates in New York school districts. Subsequently, a multivariate ordinary least squares linear regression was conducted using sociodemographic and other relevant variables. Lastly, correlations were conducted to assess relationships between ELL status, ASD rates, race/ethnicity, economic disadvantage, reported emotional disturbance (one of 13 disability classifications per New York State regulations) [42], and overall disability rates per school district.

**Table 3:** Multiple linear regression of variables predicting ASD prevalence in New York school districts (*n* = 865)

Variables	*r*	*β*	SE *β*
English language learners^1^	0.02	−0.00	0.00
Economically disadvantaged^1^	0.13***	−0.00***	0.00
Students with disabilities^1^	0.59***	0.10***	0.00
Students with learning disabilities^1^	0.36***	−0.09***	0.01
Students with reported emotional disturbance^1^	0.41***	0.07	0.03
Male^1^	0.14***	0.00***	0.00
Female^1^	0.16***	0.00***	0.00
Hispanic^1^	0.13***	−0.00***	0.00
Black^1^	0.22***	0.00	0.00
American Indian^1^	−0.05	−0.00***	0.00
White^1^	−0.13***	−0.00***	0.00
Asian^1^	0.10***	−0.00***	0.00
*R*^2^			0.43
*F*			60.19 (12, 923)***

***p < 0.001; **p < 0.01; *p < 0.005

^1^Percent per school district

#### Figure 1: ASD prevalence in New York school districts

Using ArcMap software, all data was classified into five equal intervals and then made into a monochromatic (i.e., multiple shades of a single color), choropleth map of ASD rates per school district. Because the data distribution was normal and fell between a small range, equal interval classification seemed appropriate to highlight variations in ASD rates per school district.

**Fig. 1:**
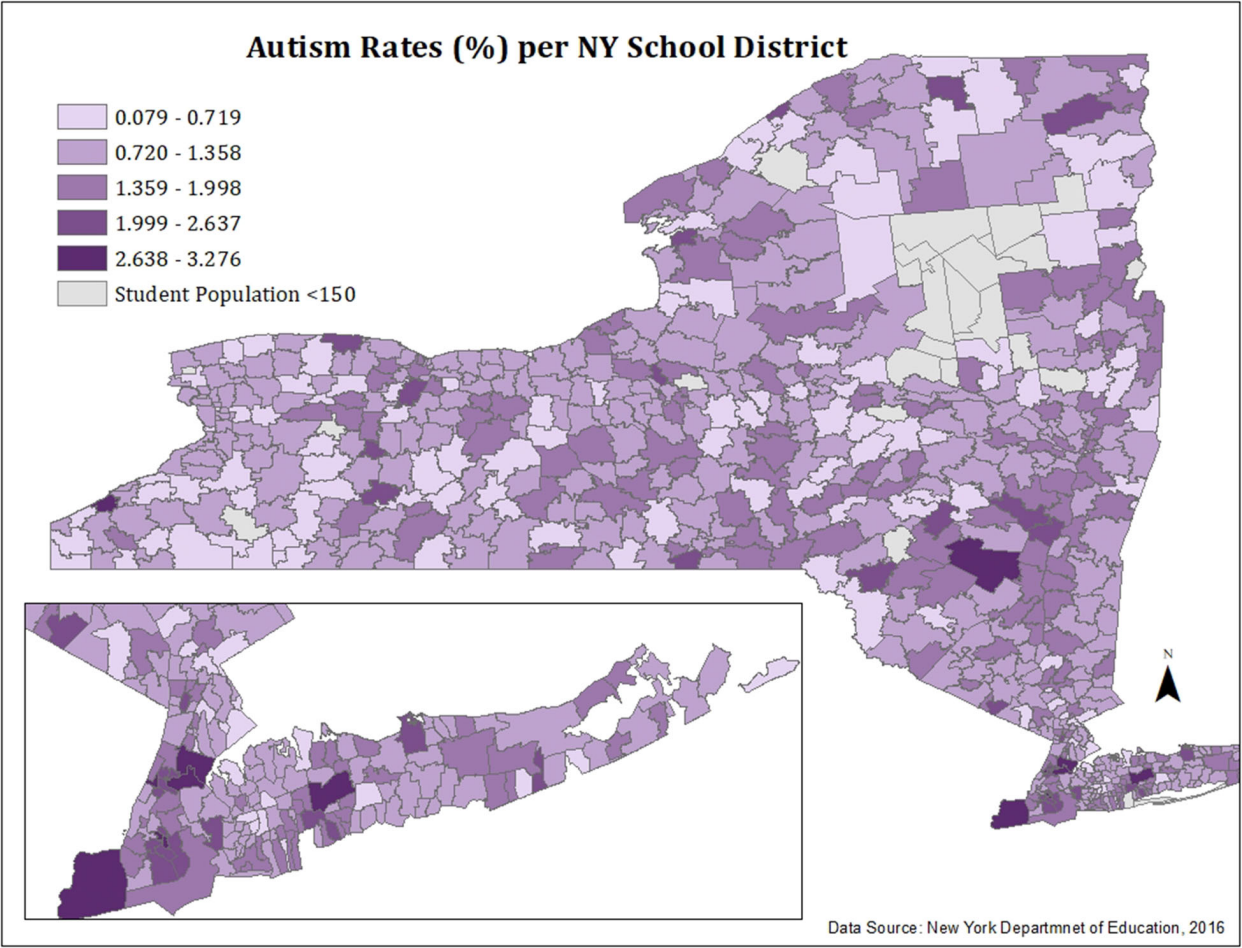
ASD prevalence in New York school districts, 2016–2017

#### Figure 2: percent ELLs per New York school district

Percentages of ELLs per school, which were mapped in ArcGIS software, ranged from 0 to 90. The distribution of this data was positively skewed, with most school districts reporting between 0 and 35% of English Language Learner students. For this reason, we felt that quantile classification would more accurately reflect the distribution of ELLs in the state.

**Fig. 2:**
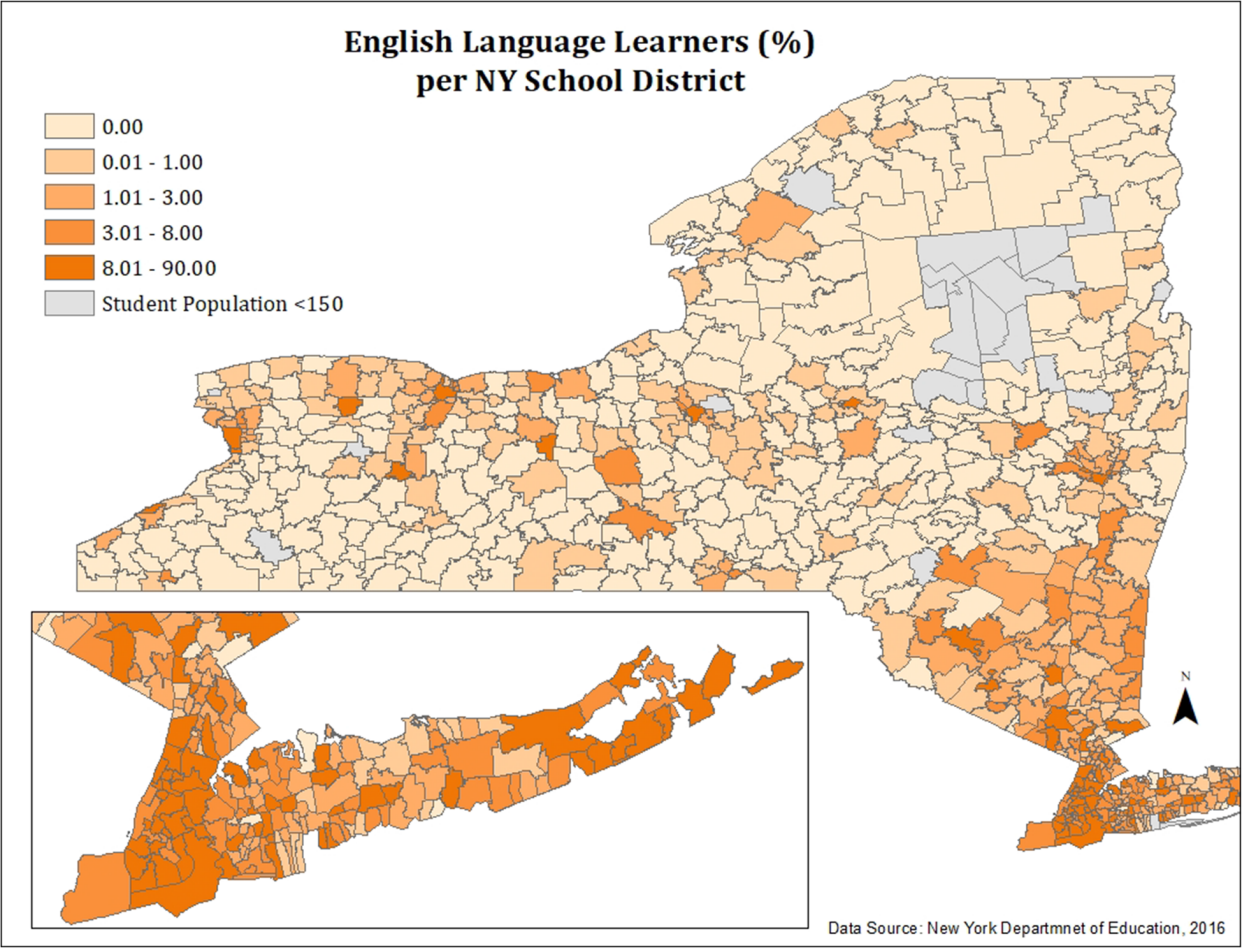
Percent ELLs in New York school districts, 2016–2017

#### Figure 3: age distribution of ASD among NYS students

Because age-specific data on ASD rates per school district were not available, we acquired an additional dataset from NYS Department of Education to explore the age distribution of students with ASD. This data provided age-specific ASD rates for the same cohort of students (those enrolled in any New York school between 2016 and 2017) used in our other analyses. Using this data, we created a bar chart to graphically display the age-specific distribution of students with ASD.

**Fig. 3:**
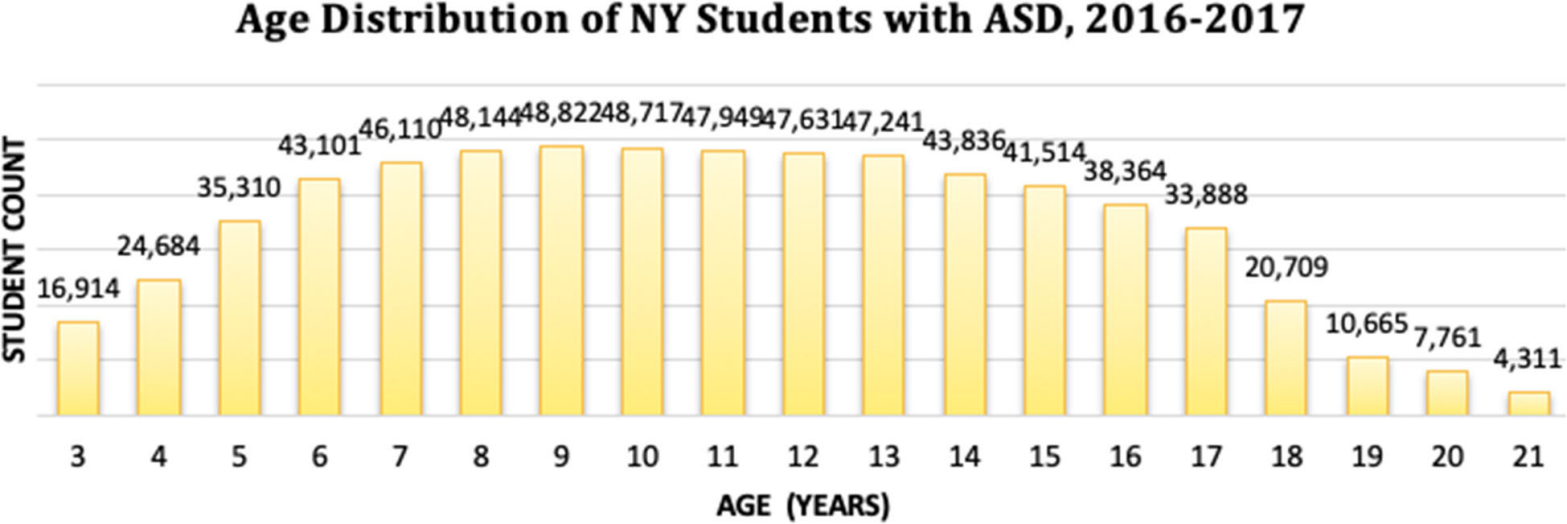
Age distribution of ASD among NYS students, 2016–2017

#### Figures 4 and 5: spatial autocorrelation (Anselin Moran’s I) of ASD and ELL rates

To test the statistical significance of spatial patterns seen in Figs. 1 and 2, spatial autocorrelation tests were performed in ArcMap. While Figs. 1 and 2 demonstrated first-order spatial effects, spatial autocorrelation was then conducted to determine second-order spatial effects. That is, spatial autocorrelation techniques were used to determine which spatial patterns occurred due to random chance and which did not. Global Moran’s I and the local Anselin Moran’s indicator of spatial autocorrelation were calculated in ArcMap for both ELL and ASD distributions.

**Fig. 4:**
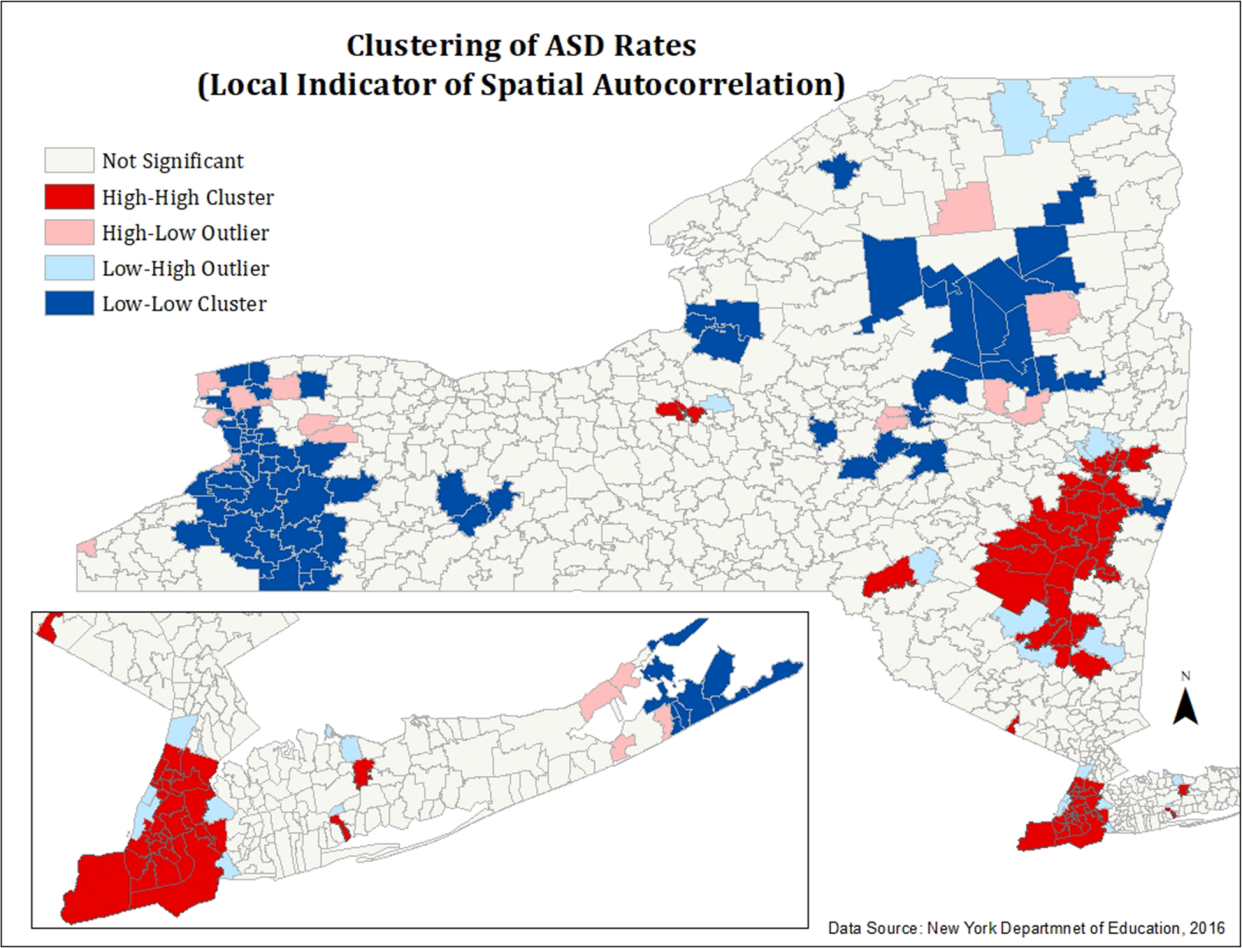
Spatial autocorrelation (Anselin Moran’s I) of ASD rates

**Fig. 5:**
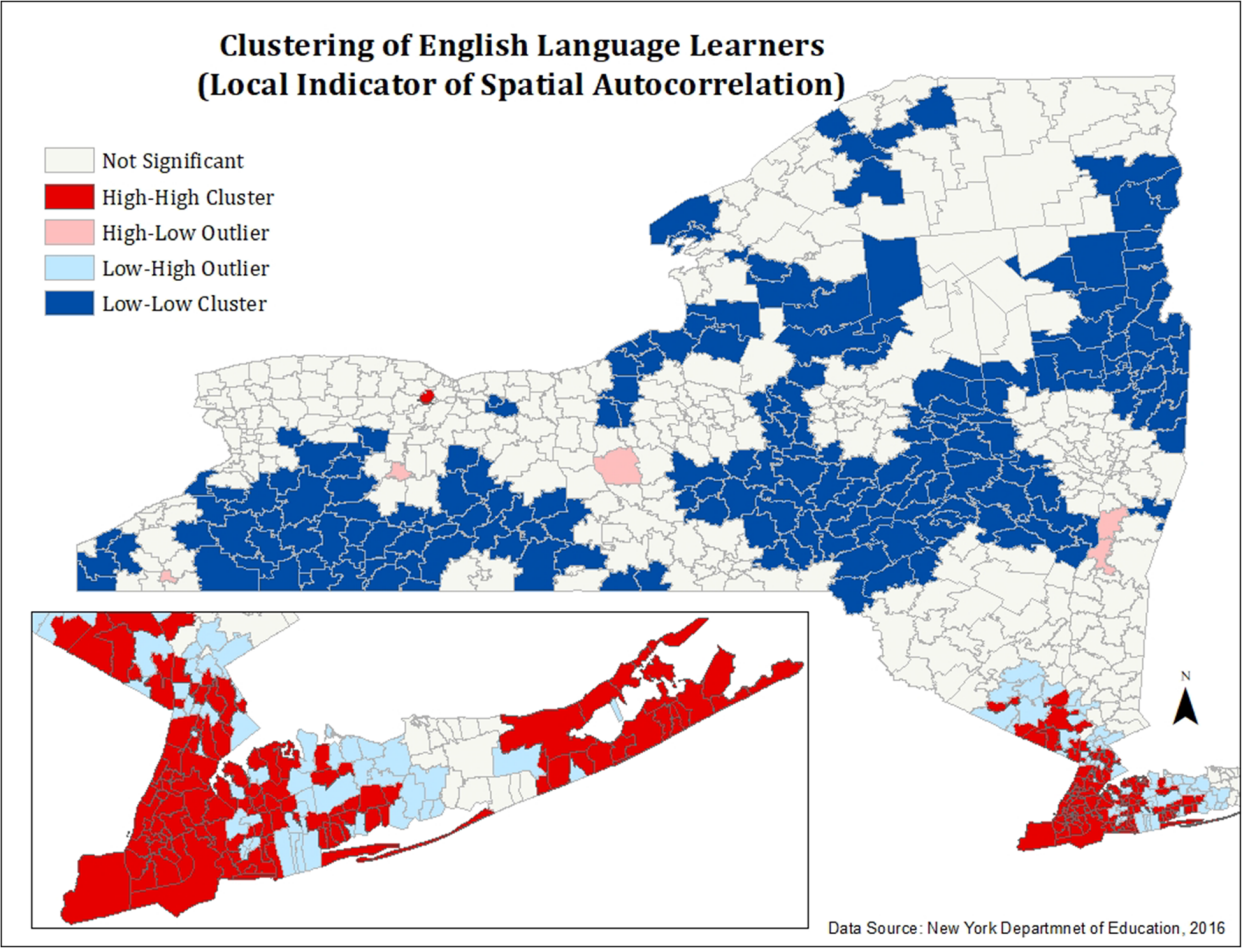
Spatial autocorrelation (Anselin Moran’s I) of ELLs

#### Figures 6 and 7 (Appendix): map of ASD Rates and ELL—standard deviations below and above mean

To provide an additional classification method and spatial portrayal ELL and ASD distributions throughout the state, maps were created in ArcMap using standard deviation classification. This classification revealed the distribution of ELL and ASD categorized by standard deviations below and above the mean.

## Results

### Sociodemographic characteristics of all school districts (Table 1)

Among the 895 school districts analyzed, there was an overall mean ASD rate of 1.14 per 100 students and an overall mean ELL percentage of 2.59. This falls within the same range as nationally reported ASD prevalence rates in the United States, which are typically between 1 and 3% [10–12]. Approximately 13.92% of all students were diagnosed with a disability (based on IDEA and New York State Special Education guidelines) and 5.21% with a learning disability. An average of 44.5% of students throughout the state was identified by the Department of Education as economically disadvantaged.

### Sociodemographic characteristics of school districts stratified by percentage of ELL students (Table 1)

Among New York school districts with the highest percentage of ELLs, there were slightly higher rates of ASD, students with disabilities, Hispanic students, and Black students, when compared to school districts with less ELL students.

### Sociodemographic characteristics of school districts stratified by percentage of ASD rates (Table 2)

Among New York school districts with the highest rates of ASD, there were 5–6% less White students, 2–3% more Hispanic students, 2–4% more Black students, and 2–4% more economically disadvantaged students, when compared to school districts with lower ASD rates. Likewise, there were higher percentages of Black and Hispanic students (approximately 2% more) in schools with the highest ASD rates, when compared against the statewide average.

Figures 1 and 2 show the spatial distribution of ASD rates and percentage of ELL students per school district. ELL students are highly concentrated around New York City, Long Island, and other urban regions of the state, including Buffalo, Utica, Rochester, Middletown, Geneva, Syracuse, and Newburgh. Except for several rural/suburban school districts, it appears that school districts with high ELL concentrations are geographically small, urban, and densely populated.

The highest ASD rates were seen among school districts located in the Bronx, Brooklyn (Kings), Manhattan (New York), Ulster, Allegany, Richmond, Greene, Suffolk, and Chautauqua counties. With the exception of one outlier—a school district in Suffolk County, Long Island—the top 25 school districts with the highest ASD rates had high percentages of economically disadvantaged (64.96%), Hispanic (18.08%), and Black (15.5%) students when compared to the sociodemographic distribution of the entire state. Additionally, these school districts also had higher rates of specific learning disabilities and IDEA categorized disabilities (i.e., specific language impairment, visual impairment, hearing impairment, etc.) when compared to the statewide average of all New York school districts.

**Fig. 6:**
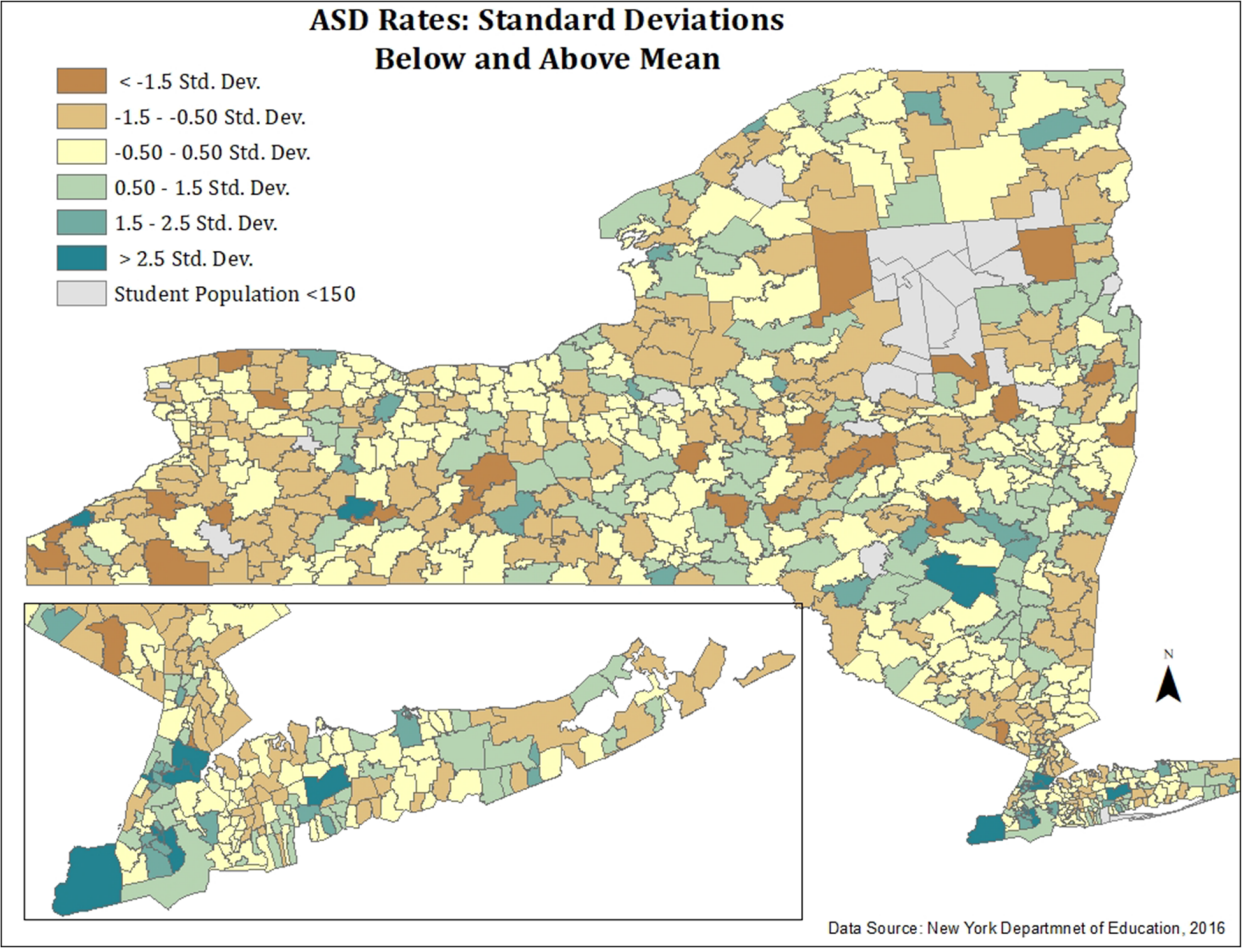
Map of ASD rates: standard deviations below and above mean

We calculated global Moran’s I and Anselin Moran’s I (Figs. 5 and 6) to confirm that the patterns of ASD and ELL distribution evidenced in Figs. 1 and 2 did not occur due to random chance.

As seen in the age distribution graph (Fig. 3) above, the number of students with ASD is lowest among those aged 3–4 and 18–21 years old. The majority of students in New York State with ASD are typically between 6 and 15 years old.

A global Moran’s index was conducted to evaluate the average spatial autocorrelation of ASD rates for the entire data set. This test revealed statistically significant, moderate, and positive autocorrelation (*I* = 0.39, *p* < 0, *z* = 17.03). A local Anselin Moran’s I was also calculated to evaluate local patterns of spatial autocorrelation among ASD diagnoses. These findings are shown in Fig. 5. Statistically significant clusters of high ASD diagnoses are concentrated in the five boroughs of New York City, but do not extend to the metropolitan regions, as shown in the previous map of ASD rates (Fig. 1). Additionally, high clusters of ASD diagnoses are seen around Albany, the state capitol.

A global Moran’s I of English Language Learners throughout the state revealed a moderate, positive spatial autocorrelation (*I* = 0.46, *p* < 0, *z* = 20.6). A local Anselin Moran’s I, shown in Fig. 6, reveals statistically significant patterns of spatial autocorrelation among English Language Learner students throughout New York State. High-high clusters (shown in red) are seen in the New York City metropolitan region, indicating that high, statistically significant clusters of English Language Learners exist within this region. Additionally, a single cluster of English Language Learners is seen in Rochester, an urban region of the state. Low-low clusters (shown in navy) of English Language Learners are dispersed throughout much of the state. Several high-low outliers (regions of high ELLs surrounded by low ELLs) are dispersed throughout the state, and multiple low-high outliers (regions of low ELLs surrounded by high ELLs) are seen outside of New York City.

### Multivariate OLS regression

As demonstrated in Table 3, correlations between ASD prevalence and ELL status, economic disadvantage, gender, race, and ethnicity were relatively weak, ranging from −0.13 to 0.22. Moderate correlations were observed between ASD prevalence per school district and students with reported emotional disturbance (*r* = 0.41) and students with disabilities (*r* = 0.59).

A univariate ordinary least squares regression was conducted to evaluate the role of English Language Learner status on ASD. The univariate model demonstrated poor model fit, *F*(1, 934) = 0.27, *p* = 0.6. Among the 895 analyzed school districts, English Language Learner status was *not* a statistically significant predictor of ASD (*B* = 0.00, SE = 0.00, *p* = 0.6). Adjusted *R*2 indicated that 0.08% of the variance in ASD prevalence is accounted for by English Language Learner status.

When economic disadvantage, disabilities, learning disabilities, emotional disturbance, gender, race, and ethnicity were added to the model, the overall fit of the model improved (*F*(12,923) = 60.19, *p* < 0.001) with an adjusted *R*^2^ = 0.43. All variables, except for percent Black and percent with emotional disturbance, were significant predictors of ASD. However, overall effect sizes were very small. This may be due to the small percentage of students with ASD per school district.

An ANOVA was conducted and revealed a significant difference (*p* < 0.001) between regression models. A post hoc Durbin-Watson test revealed that independence was not violated.

## Discussion

The primary purpose of this study was to conduct an exploratory spatial analysis of ASD prevalence among students of differing racial, ethnic, linguistic backgrounds in New York State. More specifically, we aimed to (1) explore the spatial distribution of students with ASD and ELL students in New York and (2) identify sociodemographic differences in ASD rates between ELL and non-ELL students. It is important to note, first and foremost, that this study was primarily intended to provide an *ecological and geographical perspective* on the distribution of students with ASD and English Language Learners in New York State. Because ASD is a relatively uncommon developmental disability (typically between 1 and 3% in the United States) [10–12], the sample size used in our statistical analyses was small. For this reason, correlations and OLS regression results should be interpreted with caution. However, geographic findings (specifically the geographic distribution of ELL students and students with ASD) can be interpreted at face value. These geographic findings, in addition to the sociodemographic patterns described, are exploratory and descriptive in nature. Causation cannot be assumed. Nevertheless, we will discuss some possible reasons for our findings, in addition to suggestions for future research, below.

### Aim 1: spatial distribution of students with ASD and ELL students in New York

In order to visualize the geographic distribution of students with ASD and ELL students in New York, we created two monochromatic maps of ASD rates and ELL percentages (Figs. 1 and 2) in all New York school districts using 2016–2017 data from NYS Department of Education. Additionally, we conducted further tests of spatial autocorrelation (Figs. 4 and 5) to assess for statistical significance of these observed clusters. All educational data analyzed in this study was for students aged 3–21 enrolled in nearly all public and non-public schools in New York State. This included all public, BOCES, Article 81, approved private, charter, Special Act, state supported, state acted, state agency, and out of state schools where NY residents attended.

#### Spatial distribution of school districts with highest ELL rates

Clustering of school districts with high rates of ELL students was observed on Long Island, New York City, and urban regions throughout the state. Aside from several suburban and rural outliers, districts with high ELL density tended to be geographically compact, densely populated, and urban. Spatial autocorrelation tests revealed statistically significant clustering of school districts with high ELL rates in the New York City metropolitan area and Rochester.

#### Spatial distribution of school districts with highest ASD rates

Similar to districts with the highest rates of ELL students, school districts with the highest rates of ASD were also concentrated in densely populated, urban, and geographically smaller school districts. School districts with higher densities of students with ASD were clustered around New York City and Albany, the state capitol.

### Aim 2: sociodemographic differences in ASD rates among ELL and non-ELL students

Analysis of educational data for all New York school districts with student populations over 150 revealed an average ASD rate of 1.1% and ELL rate of 2.6%. Our findings on ASD prevalence in New York are consistent with national prevalence rates, which generally fall between 1 and 3% [10–12]. However, we found that the percent of ELL students in New York is considerably lower than the national average for U.S. public schools during the same year (2.6% vs. 10.1%, respectively). According to data from the Department of Education, the states with the highest percentage of ELL students in 2017 were California (19.2%), Texas (18%), Nevada (17.1%), and New Mexico (16.3%). Consistent with our study findings, the Department of Education also reported that most ELL students in 2017 resided in cities, when compared to non-urban settings (rural, suburban, and towns) [43]. Interestingly, we found that students with ASD and other learning and developmental disabilities also tended to be concentrated in urban areas.

Among school districts with the highest rates of ASD in New York (top third), there were approximately 4% *less* White students, 2% *more* Hispanic students, and 2% *more* Black students than the statewide average. These districts also had 3% more students with IDEA-defined disabilities, including deaf-blindness, deafness, emotional disturbance, hearing impairment, intellectual disability, learning disability, multiple disabilities, orthopedic impairment, other health impairment, speech-language impairment, traumatic brain injury, and/or visual impairment. Districts with the most ELLs (top third) had *slightly* higher rates of ASD than the statewide average (0.12% vs. 0.49%) and a greater composition of Asian, Hispanic, and Black students (3.7, 12.7, and 7.2%, respectively). The majority of students in districts with the highest ASD rates were economically disadvantaged. Additionally, districts with the highest rates of ASD had higher representations of Hispanic, Black, and students with any disability when compared to the statewide average.

Correlations between sociodemographic variables and ASD rates were fairly weak. However, moderate correlations were observed between ASD rates, percent of students with reported emotional disturbance, and percent of students with disabilities. Univariate regression revealed that ELL status was *not* a statistically significant predictor of ASD. When economic disadvantage, disabilities, learning disabilities, emotional disturbance, gender, race, and ethnicity were adjusted for, model fit improved and indicated a statistically significant relationship (*R*^2^ = 0.43, *p < 0*.001). Although most variables in the model were significant predictors of ASD, the effect sizes were very small. This may be due to the relatively low rate of ASD prevalence per school district. For this reason, results of our correlations and OLS regressions should be interpreted with caution. A larger sample size (i.e., from a national data set) would offer greater statistical power.

In summary, when both ASD rates and ELL percentages were stratified by low, medium, and high ranges, we found a greater representation of Black and Hispanic students among the highest tertiles. School districts with the highest rates of ASD revealed slightly lower concentrations of White students and higher concentrations of Black and Hispanic students, as well as higher concentrations of students with disabilities. Geographic analysis revealed higher proportions of Hispanic, Black, and economically disadvantaged students among districts with the highest ASD rates. These districts also had notably higher percentages of students with learning disabilities and disabilities overall. Clustering of school districts with high ASD rates was observed within New York City and Albany. Analysis of ELLs by tertiles revealed higher percentages of Asian, Black, and Hispanic students than the average statewide student population. Maps of ELLs throughout the state revealed higher concentrations of ELLs in urban regions and statistically significant clustering of high ELL percentages in New York City and Rochester. Although we anticipated greater racial and ethnic diversity among school districts with high concentrations of ELL students, we were surprised to find greater representation of Black and Hispanic students among school districts with the highest ASD rates, as historically ASD has been less diagnosed among racial and ethnic minorities in the United States.

Research on ASD diagnostic disparities among racial, ethnic, and linguistic minorities is continually evolving. National ASD prevalence data in the United States is typically pulled from one of three sources: (1) the Centers for Disease Control & Prevention ADDM Network, (2) the National Health Interview Survey, and (3) the National Survey of Children’s Health. Because of the varying sampling and methodological techniques used in each data source, true ASD prevalence can be difficult to capture. This becomes even further complicated when analyzing ASD prevalence on an smaller scale (i.e., state level) or analyzing differences among persons of varying racial, ethnic, linguistic, and geographic backgrounds. Fluctuations in ASD prevalence have also been influenced by other factors, including changes in diagnostic criteria, increased awareness of ASD, and other spatial and contextual drivers (i.e., access to services) [44, 45].

Findings from the 2020 ADDM Community Report indicate that, for the first time, the diagnostic gap between Black and non-Hispanic White children monitored in this network has closed. However, Hispanic/Latino children in the ADDM Network still remain underdiagnosed by the age of 8 when compared with peers of other races and ethnicities [46]. Additionally, delayed diagnosis still remains a concern among Black and Hispanic/Latino children, as they are still likely receiving an ASD diagnosis later than their White counterparts [46]. A recent analysis of data from the Autism Genetic Resource Exchange, the largest phenotypic and diagnostic data repository on children with ASD, found that African American children experienced a 3-year lag in ASD diagnosis from the time parents reported concerns [47]. Other national data sources still report higher rates of ASD among non-Hispanic Whites when compared to Black and Hispanic children [10, 13–33, 48]. Because early intervention is critical in reducing the severity of impairment and disability associated with a condition such as ASD, continued efforts to reduce delays in diagnosis among racial and ethnic minorities are greatly needed and should remain a top priority. Although we found a greater representation of Hispanic and Black students among New York school districts with the highest rates of ASD (contrary to most national ASD prevalence data), we suspect this may attributed to geographic factors.

Language status, in addition to race and ethnicity, can also contribute to delays in proper diagnosis of ASD or any developmental disability. The social, behavioral, and communicative patterns observed in children with ASD (i.e., delayed language acquisition, challenges with socialization) can be a *normative* process of second language acquisition among ELLs [49–51]. Delays in reaching certain developmental milestones or language development among ELL students are not necessarily indicative of an underlying learning or developmental disability. Further research on differentiating normal second-language acquisition from developmental disability is greatly needed. Because of the sparsity of research on this topic, as well the predominant use of diagnostic measures normed from European-American populations, ELLs remain overrepresented in the United States’ special education system [52]. To avoid delayed or inaccurate diagnosis of ASD or other disabilities among ELLs, it is recommended that health and school professionals allow for flexibility in assessment measures (i.e., multimethod informant interviews, use of non-language dependent assessment tools, assessment in the child’s primary language, and less stringent time requirements), consideration of the child’s home and language environment, and deep attunement to the culture of both the child and family [49–53].

In our sociodemographic and geographic analyses, we also observed that most ELL students and students with ASD were clustered in urban regions of the state. Prior research has suggested that there may be potential associations between urbanicity and ASD. A study of a Danish cohort of over 800,000 children found that greater urbanicity was associated with higher risk of ASD. This study took into account both children’s residence at birth and residence during childhood [54]. Researchers from the Taiwan Birth Cohort Study, which featured a study sample of 20,095 children, also found that there were more children with ASD located in urban areas [55]. Although various environmental and etiological factors may influence the association between ASD and urbanicity, one potential thought is that proper diagnosis and access to diagnostic services are more readily available in urban areas. Higher rates of ASD, as well as greater utilization of specialty disability services, have been observed in the northeast regions of the United States [56, 57]. Within the United States, ASD diagnostic facilities and resources are unevenly distributed. Alarmingly, a recent study found that nearly 84% of all counties had no ASD diagnostic facility at all, and that among counties with adequate facilities, wait times were very long [58]. This highlights the glaring need for efficient, high quality, evenly distributed ASD services in the United States. Additionally, further research on available services for ELLs is also greatly needed.

## Conclusion

The findings from this study are intended to provide a snapshot into the sociodemographic composition and geographic distribution of ELL students and students with ASD in New York. Contrary to previous literature on ASD prevalence, which indicates that ASD is underdiagnosed among persons from racial, ethnic, and linguistic minority groups and lower socioeconomic status, we observed a higher concentration of Black, Hispanic, and economically disadvantaged students among school districts with the highest percentages of ASD. Additionally, we observed higher rates of learning disabilities and overall disabilities among school districts with the highest ASD rates. We were surprised to see overall higher rates of disability among school districts with greater proportions of minority students and students of lower socioeconomic status. This could potentially indicate greater awareness of, or training in, learning disabilities in high-need school districts in New York State. Also, because school districts with higher ASD rates tend to be concentrated in New York City and Albany, it may be possible that these students have greater access to diagnostic services, leading to higher rates of diagnosis within the school district. We were also interested to find that many English Language Learners are often concentrated in urban areas and more densely populated school districts of smaller geographic size. Because of the exploratory nature of this study, we can only *describe* sociodemographic associations with ASD and ELLs but cannot assume causality by any means.

This study presented with several limitations. For one, school district data is but one method of capturing ASD diagnoses and does not necessarily provide a comprehensive snapshot of actual ASD prevalence. Because of the overall rarity of ASD, and hence possible risk of student identification, we analyzed ASD rates at a school district, rather than individual, level. Analysis of individual level data on a larger scale (i.e., national) would provide a more accurate portrayal of ASD distribution. Nevertheless, school district data does offer large statistical power and provides insight into sociodemographic variables that may be associated with ASD diagnosis. To improve our national and state developmental disability surveillance systems, it may be beneficial to further develop a unified data collection system across all states that utilizes both school and health records. Another major limitation of our study is that we did not use age- or grade-stratified data. Because delayed diagnosis among racial, ethnic, and linguistic minorities is such a pertinent issue, it would be highly beneficial to see how age of diagnosis varies among differing groups of students, including ELLs. Additionally, it may also be beneficial to further explore how urban, suburban, and rural classifications may impact the distribution of students with ASD and ELL students outside of New York. This may help to identify regions with the greatest need for disability diagnostic and treatment services. Lastly, we recommend that further research be dedicated to understanding the unique needs and experiences of ELL students. It is hoped that increased understanding of a child’s home and language environment, flexibility in diagnostic assessment and treatment, and attunement to a family’s culture will help avoid underdiagnosis or misdiagnosis of ASD and other developmental disabilities.

**Fig. 7:**
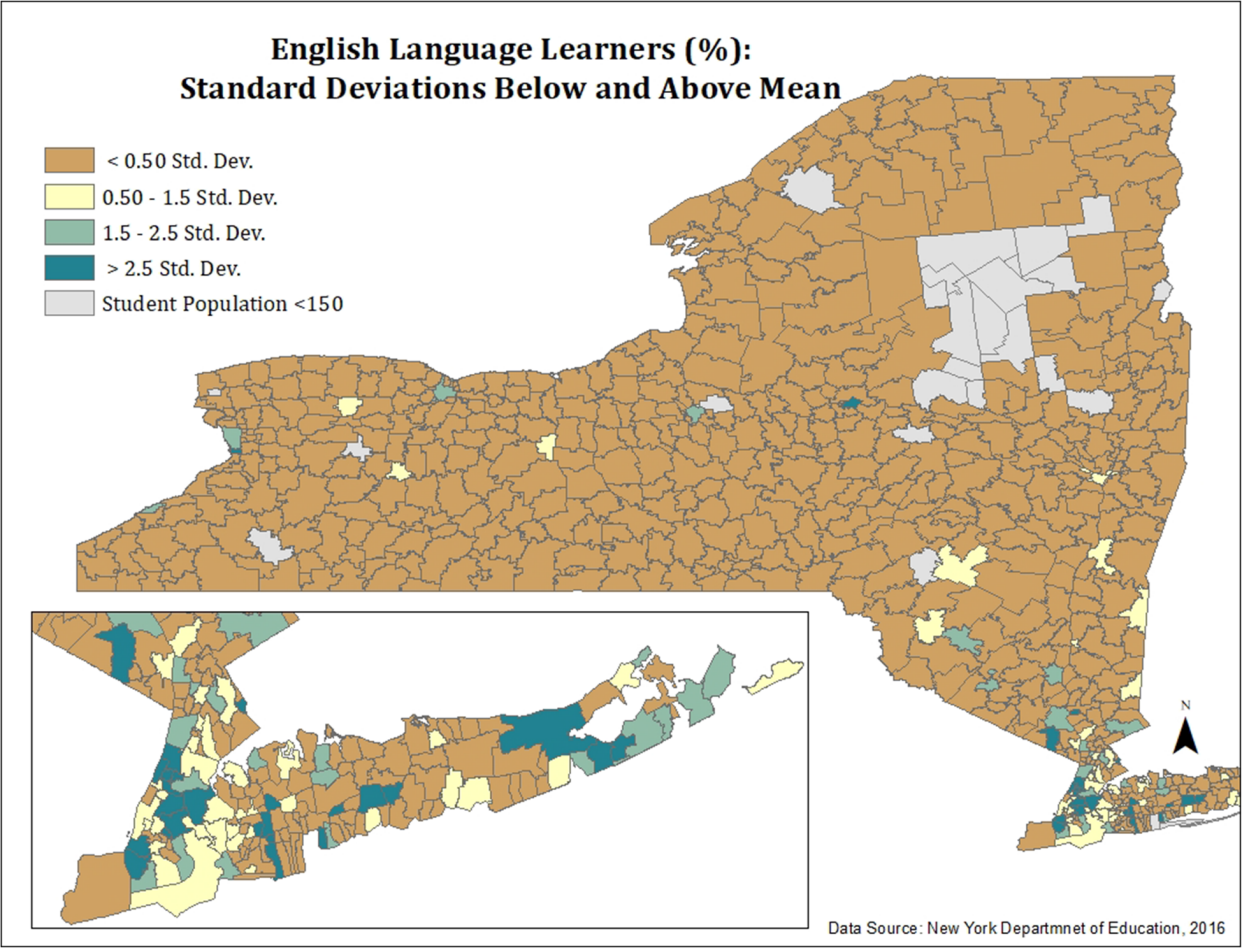
Map of ELL students: standard deviations below and above mean. Figures 6 and 7 highlight percentages of ASD and ELL per school district, respectively, by standard deviations below and above the mean for each characteristic. Figure 3 reveals similar findings to ASD rates (Fig. 1), especially for districts with the highest and lowest rates of ASD. School districts with the highest rates of ASD are highlighted in dark green (> 2.5 SD above the mean). As with those in previous figures, school districts with the highest ASD rates (> 2.5 SD above the mean) also possessed higher percentages of economically disadvantaged, Black, and Hispanic students. When stratified by standard deviations, ASD rates did not reveal a clear geographic pattern, except for some clustering of high ASD rates around city centers (i.e., New York City). In contrast, ELL distribution (Fig. 4) presents a clearer pattern with nearly all districts classified into the < 0.50 SD below the mean (dark orange) category, in contrast to the New York City metropolitan area where the percent of ELLs is > 2.5 SD above the mean (dark orange).

## Data Availability

All data is publicly available and can be accessed from the following URLs: 1) New York State Department of Education (2018). Number of NYS children and youth with disabilities receiving Special Education programs and services as of October 5, 2016. Retrieved from: http://www.p12.nysed.gov/sedcar/state.htm 2) New York State Department of Education (2018). Enrollment database. Retrieved from: https://data.nysed.gov/downloads.php 3) New York State Geographic Information Systems Program Office (2018). GIS set details: NYS Schools and School District Boundaries. Retrieved from: https://gis.ny.gov/gisdata/inventories/details.cfm?DSID=1326 4) United States Department of Education (2020). Individuals with Disabilities Education Act data. Retrieved from https://sites.ed.gov/idea/data/
